# Supplementary material for: A Novel In Vivo Model for Multiplexed Analysis of Callosal Connections upon Cortical Damage
Source: Int J Mol Sci. 2022 Jul 26;23(15):8224. doi: 10.3390/ijms23158224 (PMC9368090; doi:10.3390/ijms23158224)
Supplement: Supplementary file 1 [file ijms-23-08224-s001.zip › 202207_Supplemental figures_ijms-1821574.pdf]

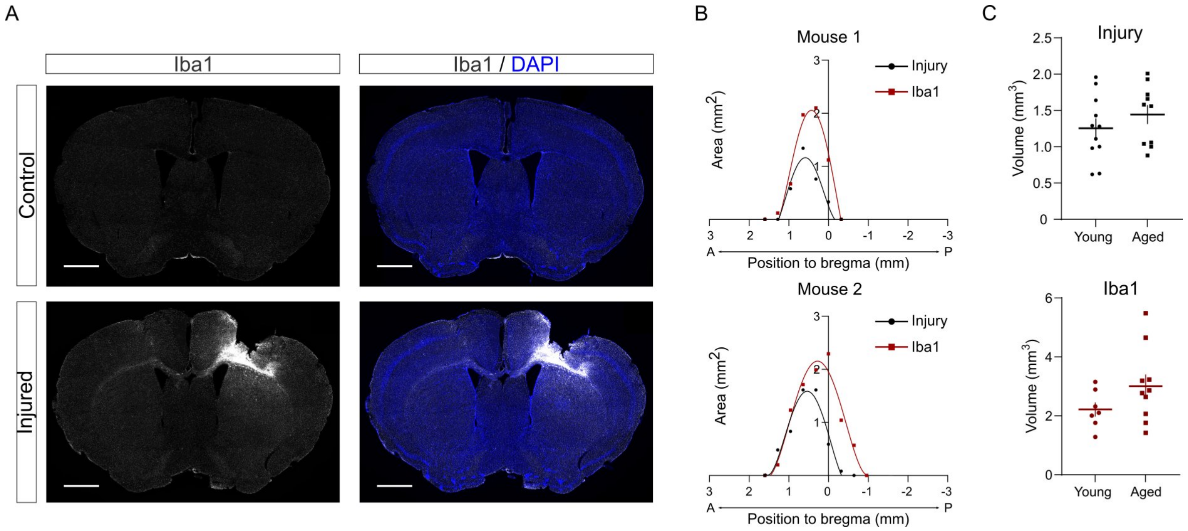

Supplemental Figure S1. Injury and inflammation in young mice upon CCD.

(A) Representative images of control and injured young animals (4 months) labeled for Iba1 marker upon CCD. Scale bar, 1mm.

(B) Cartesian graphs represent data of injury (black) and Iba1 (red) staining areas of two different young animals, positioned relative to bregma coordinates.

(C) Graph shows the volume quantification of the injury (top; p=0.33; unpaired t test) and Iba1 staining (bottom; p=0.14; unpaired t test) in injured young and aged mice. Iba1, n=7 young animals, n = 10 aged animals; Injury, n=11 young animals, n= 10 aged animals. Average ± sem.

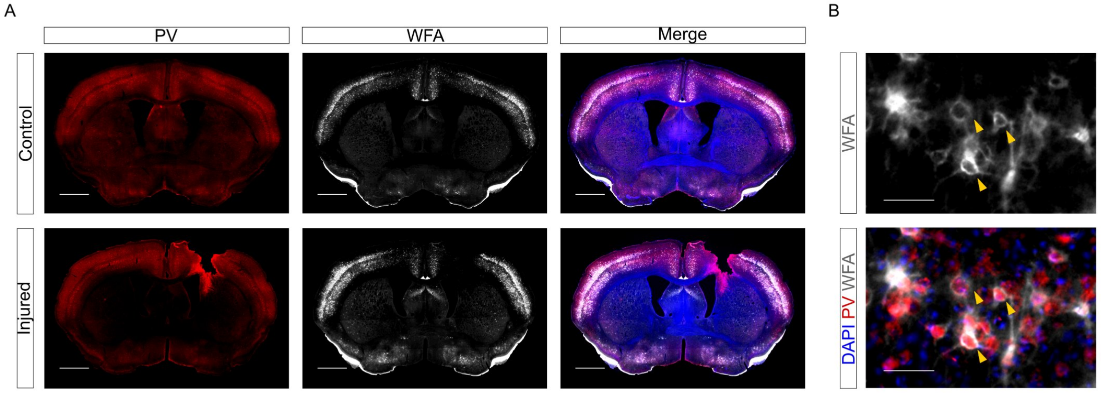

Supplemental Figure S2. WFA+ PNNs surround PV+ interneurons.

(A) Representative images of PV and WFA staining in control and injured mice. Scale bar, 1mm.

(B) Magnified areas from an injured sample are represented to show PV and WFA staining in detail. Yellow arrows indicate the co-localization of PV+ interneurons and WFA+ PNNs. Scale bar, 50μm.
