# Supplementary material for: A Novel In Vivo Model for Multiplexed Analysis of Callosal Connections upon Cortical Damage
Source: Int J Mol Sci. 2022 Jul 26;23(15):8224. doi: 10.3390/ijms23158224 (PMC9368090; doi:10.3390/ijms23158224)
Supplement: Supplementary file 1 [file ijms-23-08224-s001.zip › SUPPLEMENTAL MATERIAL_S1_Behavioral analysis.pdf]

Supplemental Material S1: Score criteria for Motor characterization of CCD model

Table S1. Rotating pole test (Talhada, *et al*. 2019)	
**Score**	**Criteria**
0	Animal falls off immediately upon entry onto the pole
1	Animal remains embraced to the pole unable to cross and eventually falls off the pole
2	Animal falls off during crossing or if the hind limbs do not contribute to forward movement
3	Animal crosses the pole while continuously slipping with the fore- or hind limbs
4	Animal crosses pole with more than 3ft slips
5	Animal traverses the pole with 1-3 foot slips
6	Animal crosses the pole without any foot slips.

Table S2. Narrow/Wide-spaced ladder	
**Score**	**Criteria**
1	Impossible to walk
2	Asymmetric walking pattern, more than 7 mistakes
3	Asymmetric walking pattern, more than 4 mistakes
4	Slightly asymmetric walking pattern, 2-4 mistakes
5	Perfect walking

Table S3. Treadmill	
**Score**	**Criteria**
1	Impossible to walk
2	Difficulties in coordination, clearly unbalanced
3	Obviously impaired and unbalanced
4	Slightly impaired and unbalanced
5	Perfect walking

Tables S1-S3. Scoring system used for the rotating pole (Table 1), narrow- and wide-spaced ladder (Table 2) and treadmill (Table 3) to evaluate the functional motor outcome performed by the aged mice before, D2 and D7 after CCD.

Table S4. Detection settings	
Camera gain (dB)	7.4
Green Intensity Threshold	0.12
Red Ceiling Light (V)	17.7
Green Walkway Light (V)	16

Table S5. Run criteria	
Minimun run duration (s)	0.55
Maximun run duration (s)	4
Minimun number of compliant runs to acquire	10
Use maximum allowed speed variation (%)	60

Tables S4-S5. Experimental options of Catwalk XT software to adjust detection settings (Table 4) for an appropriate animal recording and run criteria (Table 5) to filter valid runs for further automatic analysis.
