# Supplementary material for: A Novel In Vivo Model for Multiplexed Analysis of Callosal Connections upon Cortical Damage
Source: Int J Mol Sci. 2022 Jul 26;23(15):8224. doi: 10.3390/ijms23158224 (PMC9368090; doi:10.3390/ijms23158224)
Supplement: Supplementary file 1 [file ijms-23-08224-s001.zip › SUPPLEMENTAL MATERIAL_S2_Python code.pdf]

Supplemental Material S2: Python code for sorting the image stacks:

Requirements:

This automatic process was written in the Python language. For the execution of this code, we have used a python installation in version >3.9 and we have installed the tifffile and skimage libraries that are not included in the default installation. To simplify this task, Anaconda v2021.11 software was installed, facilitating the execution environment in the current operating systems (Windows, Linux and MacOS).

When executing the code from the terminal, it is executed with the following arguments:

+ python3 ordered_stack.py DIRECTORY_OF_MOUSES

This argument must be the folder where the mouse folders with the tif files of the region of interest are stored. This would be an example of the structure:

```
Project
├── Mouse_384
│   ├── AGM52_384_DAPI_mask.tif
│   ├── AGM52_384_DAPI_stack.tif
│   ├── AGM52_384_GFAP_stack.tif
│   ├── AGM52_384_GFP_stack.tif
│   └── AGM52_384_Iba1_stack.tif
├── Mouse_389
│   ├── AGM52_389_DAPI_mask.tif
│   ├── AGM52_389_DAPI_stack.tif
│   ├── AGM52_389_GFAP_stack.tif
│   ├── AGM52_389_GFP_stack.tif
│   └── AGM52_389_Iba1_stack.tif
├── Mouse_393
¦   ¦
¦
```

Taking special care with the nomenclature of the files as they have to have a specific format for their correct functioning. A scheme would be "subject_id_type_orig-or-mask.tif" where:

+ subject_id: is our subject identifier.

+ type: is the type of file we have (DAPI, GFPA, GFP and Iba1) For the code to work, all 4 files must be present.

+ orig-or-mask: Indicates if it is the original image stack or a mask. In this case, we will only have a mask for the DAPI version.

<u>Script:</u>

```
import sys

import numpy as np

import cv2

import tifffile

import os

from skimage import io

from skimage import morphology as morph

from pathlib import Path
```

# It goes through all the folder of the mice. NOTE: "glob" parameter distinguish between upper and lower case letters

```
for subject_path in sorted(Path(sys.argv[1]).glob("*Mouse*")):

    print(f"processing mouse {subject_path.name}")

    # Create a forder where png results will be stored

    png_results = subject_path.joinpath("png_results")

    os.makedirs(str(png_results), exist_ok=True)
```

# For each mouse folder, it lists the directory. The code saves all objects in the folder, regardless of whether it is a **folder or an image**

```
    stacks = list(subject_path.iterdir())
```

# It sees if there is a file in the stack that says "mask"

```
    if any(["mask.tif" in str(file_) for file_ in stacks]):

        # Nos guarda los paths de cada imagen

        dapi_path = [file_ for file_ in stacks if "DAPI_stack.tif" in str(file_)][-1]

        mask_path = [file_ for file_ in stacks if "DAPI_mask.tif" in str(file_)][-1]

        red_path = [file_ for file_ in stacks if "GFAP_stack.tif" in str(file_)][-1]

        green_path = [file_ for file_ in stacks if "GFP_stack.tif" in str(file_)][-1]
```

farred_path = [file_ for file_ in stacks if "Iba1_stack.tif" in str(file_)][-1]

# Image upload

dapi = np.array(io.imread(str(dapi_path)))

mask = np.array(io.imread(str(mask_path)))

red = np.array(io.imread(str(red_path)))

green = np.array(io.imread(str(green_path)))

farred = np.array(io.imread(str(farred_path)))

# It creates a list with the size of the number of slices of the mask, save the area it takes up

feret_area_list = np.zeros(mask.shape[0])

# It scrolls through each slice of the mask stack

for image_ind in range(mask.shape[0]):

# It saves a png file with the original mask

cv2.imwrite(

str(png_results.joinpath(f"{dapi_path.stem}_normal_mask_{image_ind}.png")), mask[image_ind]

)

# Within the stack, it removes noise (e.g. speckles around the stack, background removal, debris around the slice)

area_open = morph.area_opening(mask[image_ind], area_threshold=40000)

# It saves a png with the mask processed with "area_opening"

cv2.imwrite(

str(png_results.joinpath(f"{dapi_path.stem}_remove_small_objects_{image_ind}.png")), area_open

)

# It eliminates vertexes of the brain, outline the contours of the brain

kernel = cv2.getStructuringElement(cv2.MORPH_ELLIPSE, (7, 7))

area_open = cv2.erode(area_open, kernel, iterations=5)

save a png with the mask processed with "erode"

cv2.imwrite(str(png_results.joinpath(f"{dapi_path.stem}_E_{image_ind}.png")), area_open)

# It rebuilds what has been eroded. NOTE: x2 is done so that the separate slices at the beginning are better sorted. If this does not work, you can use x3

area_open = cv2.dilate(area_open, kernel, iterations=5)

save a png with the mask processed with "dilate"

cv2.imwrite(str(png_results.joinpath(f"{dapi_path.stem}_ED_{image_ind}.png")), area_open)

area_open = cv2.dilate(area_open, kernel, iterations=5)

# It saves a png with the mask processed with "dilate"

cv2.imwrite(str(png_results.joinpath(f"{dapi_path.stem}_EDD_{image_ind}.png")), area_open)

Finds the contours of the mask to be able to apply the convexHULL

contours, hier = cv2.findContours(area_open, cv2.RETR_EXTERNAL, cv2.CHAIN_APPROX_SIMPLE)

# It transforms the mask from grayscale to colour in order to display the result of convexHULL

color_img = cv2.cvtColor(area_open, cv2.COLOR_BGR2RGB)

# For each contour found in the image. Note: there should be only 1, but it may be the case that the sample is split

for contour_ind in range(len(contours)):

convex HULL: creates an outline of the object by skipping the convex parts

hull = cv2.convexHull(contours[contour_ind], returnPoints=True)

Calculate the area

area = cv2.contourArea(hull)

Save area

feret_area_list[image_ind] += area

color_img = cv2.addWeighted(

```
            color_img, 1., cv2.drawContours(color_img, [hull], 0, (100,240, 230), 7), 1., 0
        )
```

# It saves a png with the contour calculated with "convexHull" and the mask

```
        cv2.imwrite(str(png_results.joinpath(f"{dapi_path.stem}_hull_{image_ind}.png")),
color_img)
```

# It rearranges the cuts according to the calculated area

```
    dapi_ord = dapi[np.argsort(feret_area_list), ...]
    mask_ord = mask[np.argsort(feret_area_list), ...]
    red_ord = red[np.argsort(feret_area_list), ...]
    green_ord = green[np.argsort(feret_area_list), ...]
    farred_ord = farred[np.argsort(feret_area_list), ...]
```

# It saves the results in new tif files

```
    tifffile.imwrite(
        dapi_path.parent.joinpath(dapi_path.stem + "_ord" + ".tif"),
        dapi_ord,
        imagej=True
    )
    tifffile.imwrite(
        mask_path.parent.joinpath(mask_path.name + "_ord" + ".tif"),
        mask_ord,
        imagej=True
    )
    tifffile.imwrite(
        red_path.parent.joinpath(red_path.name + "_ord" + ".tif"),
        red_ord,
        imagej=True
    )
```

```
# tifffile.imwrite(
#     green_path.parent.joinpath(green_path.name + "_ord" + ".tif"),
#     green_ord,
#     imagej=True
# )
tifffile.imwrite(
    farred_path.parent.joinpath(farred_path.name + "_ord" + ".tif"),
    farred_ord,
    imagej=True
)
```
