# Supplementary material for: A Novel In Vivo Model for Multiplexed Analysis of Callosal Connections upon Cortical Damage
Source: Int J Mol Sci. 2022 Jul 26;23(15):8224. doi: 10.3390/ijms23158224 (PMC9368090; doi:10.3390/ijms23158224)
Supplement: Supplementary file 1 [file ijms-23-08224-s001.zip › SUPPLEMENTAL MATERIAL_S3_Fiji code.pdf]

Supplemental Material S3: Fiji code for stack alignment

The following examples of IJ1 macros were written for stacks of 6 and 10 slices. If you work with smaller or bigger stacks, please customize the number of iterations according to the number of slices in the stack.

Script:

Example 1: stack with 6 slices

# Rename and split loop

sk=getTitle()

run("Stack to Images");

run("Images to Stack", "name= title=[]");

run("Stack to Images");

# Select and ROI. Stack with 6 slices.

# NOTE: please, customize this part depending on the number of slices in the stack.

selectWindow("-0001");

roiManager("Select", 0);

selectWindow("-0002");

roiManager("Select", 1);

selectWindow("-0003");

roiManager("Select", 2);

selectWindow("-0004");

roiManager("Select", 3);

selectWindow("-0005");

roiManager("Select", 4);

selectWindow("-0006");

roiManager("Select", 5);

#Align save to target, defined as "trg". NOTE: xxx is the selected target. NOTE2: please, customize this part depending on the number of slices in the stack. An example is offered below for a stack of 6 samples.

```
trg= getString ("Target image, xxxx", 0003);
st1= "source=-0001 target=-"+trg+" rotate";
run("Align Image by line ROI", st1);
st2= "source=-0002 target=-"+trg+" rotate";
run("Align Image by line ROI", st2);
st3= "source=-0003 target=-"+trg+" rotate";
run("Align Image by line ROI", st3);
st4= "source=-0004 target=-"+trg+" rotate";
run("Align Image by line ROI", st4);
st5= "source=-0005 target=-"+trg+" rotate";
run("Align Image by line ROI", st5);
st6= "source=-0006 target=-"+trg+" rotate";
run("Align Image by line ROI", st6);
run("Images to Stack", "name=SkA title=aligned");
run("8-bit");
```

Example 2: stack with 10 slices

```
# Rename and split loop
sk=getTitle()
run("Stack to Images");
run("Images to Stack", "name= title=[]");
run("Stack to Images");
# Select and ROI. Stack with 6 slices.
# NOTE: please, customize this part depending on the number of slices in the stack.
selectWindow("-0001");
roiManager("Select", 0);
selectWindow("-0002");
roiManager("Select", 1);
```

```
selectWindow("-0003");
roiManager("Select", 2);
selectWindow("-0004");
roiManager("Select", 3);
selectWindow("-0005");
roiManager("Select", 4);
selectWindow("-0006");
roiManager("Select", 5);
selectWindow("-0007");
roiManager("Select", 6);
selectWindow("-0008");
roiManager("Select", 7);
selectWindow("-0009");
roiManager("Select", 8);
selectWindow("-0010");
roiManager("Select", 9);
```

#Align save to target, defined as "trg". NOTE: xxx is the selected target. Stack with 10 slices.

# NOTE: please, customize this part depending on the number of slices in the stack.

```
trg= getString ("Target image, xxxx", 0003);
st1= "source=-0001 target=-"+trg+" rotate";
run("Align Image by line ROI", st1);
st2= "source=-0002 target=-"+trg+" rotate";
run("Align Image by line ROI", st2);
st3= "source=-0003 target=-"+trg+" rotate";
run("Align Image by line ROI", st3);
st4= "source=-0004 target=-"+trg+" rotate";
```

```
run("Align Image by line ROI", st4);
st5= "source=-0005 target=-"+trg+" rotate";
run("Align Image by line ROI", st5);
st6= "source=-0006 target=-"+trg+" rotate";
run("Align Image by line ROI", st6);
st7= "source=-0007 target=-"+trg+" rotate";
run("Align Image by line ROI", st7);
st8= "source=-0008 target=-"+trg+" rotate";
run("Align Image by line ROI", st8);
st9= "source=-0009 target=-"+trg+" rotate";
run("Align Image by line ROI", st9);
st10= "source=-0010 target=-"+trg+" rotate";
run("Align Image by line ROI", st10);
run("Images to Stack", "name=SkA title=aligned");
run("8-bit");
```
